# Do Pomegranate Hydrolyzable Tannins and Their Derived Metabolites Provide Relief in Osteoarthritis? Findings from a Scoping Review

**DOI:** 10.3390/molecules27031033

**Published:** 2022-02-03

**Authors:** Marco Govoni, Francesca Danesi

**Affiliations:** 1 Reconstructive Orthopedic Surgery and Innovative Techniques—Musculoskeletal Tissue Bank, IRCCS Istituto Ortopedico Rizzoli, Via G.C. Pupilli 1, 40136 Bologna, Italy; marco.govoni@ior.it; 2 Human Nutrition Unit, Department of Agricultural and Food Sciences (DISTAL), University of Bologna, Piazza Goidanich 60, 47521 Cesena, Italy

**Keywords:** osteoarthritis, hydrolyzable tannins, ellagitannins, ellagic acid, urolithins, *Punica granatum*, gallic acid, *Mangifera indica*, chondrocytes, inflammation

## Abstract

Osteoarthritis (OA) is the most common form of arthritis affecting both the elderly and the middle-aged population. Although various therapeutics have been developed to arrest the structural deterioration of cartilage, the current treatments are limited to delay the progress of OA clinically. Therefore, it is pivotal to study new therapeutic agents for chondroprotection and the prevention of cartilage degeneration. Hydrolyzable tannin (HT)-containing foods aroused considerable interest in recent years for their relevant anti-inflammatory effects. The focus of this scoping review is to provide an overview of the evidence of the therapeutic potential of HTs and their metabolites in preventing or alleviating the course of OA. A broad search of PubMed and Scopus databases on this topic resulted in 156 articles. After the exclusion of reviews and not relevant records, 31 articles were retrieved. Although only some papers did not consider the biotransformation of HTs, most recent studies also have investigated the effect of HT metabolites. Further larger clinical trials, with an in-deep analysis of HT metabolization, are still needed to unravel the potential benefits of these compounds in OA, paving the way towards the development of a dietary strategy for the improvement of pro-inflammatory cytokine-induced chondrocyte dysfunctions and injuries.

## 1. Introduction

Osteoarthritis (OA) is the most common form of arthritis caused by joint degeneration resulting in joint pain and dysfunction [1]. Although in most instances osteochondral lesions or joint degeneration develop in the absence of an identifiable cause, mechanical stress derived by obesity, joint instability, excessive joint loading, or trauma, can promote the process of cartilage erosion and the consequent anomalous remodeling of joint tissues [2]. Besides, since the increasing longevity and the progression of large cohorts into old age are driving up the shares of the elderly around the world [3], the frequency and chronicity of OA make this syndrome the second leading cause of work disability, as well as a substantial economic burden for patients and health care systems. The Global Burden of Diseases, Injuries, and Risk Factors Study 2017 [4] reported that OA affected 303 million people globally in 2017, with an incidence of 203 cases per 10,000 person-years only for knee OA [5]. Moreover, worldwide, the estimated incidence of OA has varied from a low of 14.6 per 1000 person-years in Canada [6] to a high of 40.5 per 1000 person-years in the UK [7].

Up to the present, there is no treatment that effectively allows to arrest the structural deterioration of cartilage or restore the tissue integrity [8]. Among non-surgical procedures, although non-pharmacological strategies such as aerobic exercises, weight reduction, electromagnets, thermal modalities, or acupuncture give some improvements as for joint mobility, only pharmacological therapies show the highest efficacy in terms of pain relief, joint function, and quality of life [9].

Analgesics and non-steroidal anti-inflammatory drugs (NSAIDs) represent the first-line therapies in managing OA symptomatology [10]. However, although these drugs generally decrease pain and stiffness and improve function, they are linked to an elevated risk of tolerance and gastrointestinal toxicity, respectively.

Selective cyclooxygenase (COX) inhibitors such as rofecoxib, valdecoxib, celecoxib, or etoricoxib are preferred with respect to traditional NSAIDs for their lower incidence of gastrointestinal adverse events, whilst an increased risk of myocardial infarct compared to non-selective NSAIDs was observed, especially for long-term treatments [11,12].

Other treatment options are represented by intra-articular therapies, but their efficacy in literature remains controversial and clinical guidelines regarding their use are often inconsistent [13,14]. Glucocorticoids and hyaluronic acid are the most widely used molecules for intra-articular injections, whilst currently not categorically defined as standard-of-care [15]. Moreover, several adverse events have been reported, including hemarthrosis, synovitis, pseudogout, and muscle pain [16].

Recently, polynucleotides, a mixture of purines, pyrimidines, deoxyribonucleotides, and deoxyribonucleosides with trophic activity, have shown positive results in preclinical and clinical studies [17,18] without evidence of significant adverse events [19], albeit further investigations are needed to recognize their safety. On the other hand, most of the available pharmacological agents to relieve OA symptoms are associated with severe side effects. Therefore, it is pivotal to develop new therapies for OA, both as a safe symptomatic treatment and as an alternative therapy that would prevent cartilage lesions and degeneration.

Among non-pharmaceutical approaches for mitigating cartilage inflammation and damage, the regular consumption of (poly)phenol-rich foods with anti-inflammatory and antioxidant properties [20] should be of particular interest to professionals and practitioners that are treating OA patients willing to avoid drug toxicities and to control undesirable side-effects of conventional therapies. From this standpoint, nutrition can have a central role in both primary and secondary prevention of OA.

Improperly called “wear and tear” arthritis over time, growing evidence suggests that OA is progressing debilitating disease with a complex interaction of biomechanical and inflammatory factors. Clinical studies reported increased levels of pro-inflammatory cytokines and chemokines in the synovial fluids of patients suffering from OA of different grades [21,22] and a distinct transcriptional signature already detectable in people at risk for OA [23]. Therefore, these findings supported the rationale that joint inflammation is directly associated with OA pathogenesis and progression. In fact, in osteoarthritic states, there is a disruption of cartilage homeostasis leading to excessive production of inflammatory cytokines and matrix-degrading enzymes (collagenases and aggrecanases) and subsequent degradation of extracellular matrix (ECM) [24].

Diet may modulate inflammation, both acutely and chronically, and can play a pivotal role in the evolution of persistent low-grade inflammation, triggering or ameliorating it [25]. Emerging evidence suggests that the generation of local and systemic pro-inflammatory milieu might be one of the possible mechanisms through which unhealthy diets are linked to chronic or degenerative conditions. A recent study has demonstrated that a higher inflammatory potential of the diet is associated with an increased risk of developing knee OA in a prospective US cohort [26]. Based on that, it can be presumed that a diet rich in anti-inflammatory agents, such as a Mediterranean-style diet rich in phytochemicals, like (poly)phenols, may have a beneficial role for people at high risk of OA.

Tannins are the most complex group of (poly)phenolic compounds and can be found in edible plants either as low molecular weight (0.3 to 3 kDa) or high molecular weight molecules (up to 30 kDa) [27]. Growing evidence from in vitro and in vivo studies shows that tannins with high molecular weight have greater or more distinctive effects than low molecular weight ones [28]. Tannins can play a crucial role as a treatment for OA management, rather than as an adjunct to established pharmacotherapy, from this viewpoint.

Among tannins, the large subclass is hydrolyzable tannins (HTs) that, differently from the ubiquitous condensed tannins (proanthocyanidins), are found in relatively few species of plants. HTs are usually subdivided into two subgroups, ellagitannins (ETs) and gallotannins (GTs), whose hydrolysis yields ellagic acid (EA) and gallic acid (GA), respectively [29]. ETs are present in some fruits (pomegranates, blackberries, raspberries, strawberries), nuts (walnuts, almonds), and seeds, while mangos, pomegranates, persimmon, muscadine grapes, and some nuts are the richest dietary sources of GTs [30,31,32].

In recent years, clinical, preclinical, and in vitro studies attributed relevant anti-inflammatory properties, among others, to HT-containing foods. Pomegranate juices or extracts exhibit beneficial effects on several chronic inflammatory diseases (CIDs), such as rheumatoid arthritis, inflammatory bowel disease, and other inflammatory-associated conditions in humans and animal CID models [33,34]. Similarly, several in vivo and in vitro studies have reported the anti-inflammatory potential of other sources of HTs, such as strawberries [35] and mango juices or extracts [34,36,37].

It is well-known that HTs have a very low bioavailability [27]. Over the past few years, it became clear that the health effects of HTs were due to bioavailable molecules of low molecular weight, such as GA for GTs [38,39], EA, and urolithins–microbiota-derived metabolites–for ETs [40]. Following absorption in the gut, urolithins, EA and GA rapidly undergo phase II metabolism, occurring at the gut level and/or in the liver, which results in the release in plasma of their conjugated derivatives (*O*-glucuronidated, *O*-methylated, *O*-sulphated) that remain in circulation for a relatively long period of time before being excreted in the urine.

Interestingly, previous studies have reported some therapeutic effects of fruit (poly)phenols in mitigating inflammation of the different forms of arthritis (see Basu et al. (2018) [41] for a comprehensive review), but no clear consensus has yet emerged. Moreover, some (poly)phenols of various molecular weights, together with other bioactive compounds, were discussed or reviewed as potential dietary factors preventing or treating OA [42]. However, HTs were almost never considered despite their potential. Within this review, we discuss the findings on the potential beneficial effects on OA of HTs and HT-containing food products and evaluate their applicability for use in OA therapy. A special emphasis was laid on molecular targets involved in inflammation and the joint destruction process to understand the possible mechanisms concerning the restoration of the intra-articular environment and articular cartilage homeostasis by HTs. Finally, compared to other relevant publications in the field, this review also aims at highlighting the limitations concerning the studies here analyzed, especially for in vitro/ex vivo ones, where supra-physiological loads of HTs continue to be supplemented to cells/tissues cultured in a closed system, reaching results which are difficult to translate in vivo.

## 2. Results and Discussion

The original search yielded 156 potentially relevant publications after the exclusion of duplicates. During the screening process (reviewing of titles and abstracts), 25 records were excluded as reviews. After full-text analysis, another 100 papers were excluded. Altogether, 31 papers—ranging between 2005 and 2021—met the eligibility criteria and were selected for detailed evaluation. Specifically, six randomized clinical trials related to the HT-containing product intake in humans, 13 studies concerning the effects of consumption or treatment with HT-containing products in animal models of OA, and 12 studies on the beneficial effects of HTs or their derived metabolites assayed in cell cultures, were deeply analyzed.

### 2.1. Randomized Clinical Trials

As reported in Table 1, all listed randomized clinical trials examine the effects of HT-containing products on several endpoints such as cartilage degradation, inflammation, and pain in a population affected by knee OA.

In 2016, Ghoochani et al. (2016) [43] had published the first clinical trial aimed at evaluating the effects of pomegranate juice consumption (200 mL/day for six weeks) in patients with knee OA. Although the intake of pomegranate juice decreased the Western Ontario and McMaster Universities (WOMAC) score, improving physical function and stiffness, no significant effect on pain and matrix metalloproteinase (MMP) expression in blood samples were detected. Moreover, since authors have neither provided a placebo drink nor evaluated the concentration of MMPs in the synovial fluid, they have suggested overcoming these shortcomings by performing a longer intervention period with a double-blind placebo-controlled design, also evaluating the (poly)phenolic composition of the juice. In addition, it is worth noting that the pharmacokinetics of pomegranate ETs also deserve attention.

One year later, Rafraf et al. (2017) [44] reported for the first time the effects of a pomegranate peel extract (500 mg twice a day for six weeks) on clinical signs and symptoms of knee OA in 60 obese women, resulting in an increase of physical activity and in a reduction of joint pain. Interestingly, the same research group has evaluated the intervention on serum levels of total cholesterol and triglycerides, and it had beneficial effects on the antioxidant status of the study participants [45]. Nevertheless, these results have poor generalizability and are limited only to patients with the same demographic and anthropometric characteristics and clinical profile.

The effects of the consumption of freeze-dried strawberry powder (50 g twice a day, equivalent to about a half kilo of fresh fruits daily consumed) for 12 weeks in 17 obese patients with knee OA were investigated on pain and markers of inflammation [46] and quality of life indicators [47]. However, albeit several findings have demonstrated the role of strawberries in mitigating knee pain and reducing oxidative and inflammatory biomarkers, limitations, such as small sample size and the lack of a dose-response design, could affect the interpretation of results. Moreover, as reported by Schell et al. (2017) [46], the generalizability of these findings is complicated, especially in post-traumatic OA or in the non-obese OA population.

Together with pomegranate fruits and strawberries, also blueberries are a rich source of HTs (160 mg GA equivalents per 100 g of fresh weight, as reported by Diaconeasa et al. (2015) [49]) and are widely consumed. Du et al. (2019) [48] performed a randomized, double-blind trial to evaluate whether the regular consumption of freeze-dried blueberries (40 g/day) in 63 obese adults with knee OA might positively affect pain, gait performance, and inflammation. Although patients in the blueberry arm have achieved a significant reduction of the WOMAC score, tumor necrosis factor α (TNF-α) and the inflammatory cytokines, such as interleukin 1β (IL-1β) and interleukin 6 (IL-6), did not change compared to the placebo group. Moreover, this study was affected by a high dropout rate and an over-representation of female OA patients precluding a drawn of firm conclusions.

Although each study was differently designed, based on the here reviewed trials, the consumption of HT-rich foodstuffs or products has allowed achieving similar results in terms of improvement of physical function and stiffness, an increase of the antioxidant status, and a reduction of breakdown cartilage enzymes. It is worth noting that these beneficial effects also depend on the presence of other bioactive compounds, such as flavonoids, which are potentially contained in the different amounts into the specific extract, juice, or powder used in each study. As an example, Basu et al. (2018) [47] reported that the administered dose of 50 g strawberry powder provided approximately 1585 mg total (poly)phenols, 66 mg anthocyanins, and 220 mg EA. Unfortunately, a detailed characterization of the tested intervention product is not provided by most of the authors (Table S1), although it would be essential information to share with clinicians and researchers. Besides, the bioactivity of HTs can be influenced by their possible interactions with the food matrix. In turn, these interactions depend on the presence of other components which can enhance or antagonize HT biological activities [50]. For instance, EA shows higher bioavailability and bioactivity when taken in with pomegranate juice than as the free form [51]. Moreover, other limitations–(i) a gender restriction (i.e., female population [44,45]), (ii) the presence of a specific co-morbidity (i.e., obesity [44,45,46,47,48]), and/or (iii) the concomitant use of drug therapy [44,45]–could reduce the emphasis related to the results. Hence, there is still the need for specific guidelines, which drive the clinicians and researchers to properly design human studies bearing in mind critical factors, such as an appropriate sample size, power, and study population, which are essential for assessing the effectiveness of an intervention. However, as reported in Table 2, among the most recent clinical trials registered on Cochrane Central Register of Controlled Trials [52], only one study (i.e., NCT03703024 [53]) has enrolled a high number of patients, but results have not been published yet.

### 2.2. In Vivo OA Studies

Table 3 summarizes the studies based on the use of in vivo preclinical animal models employed to investigate the possible therapeutic efficacy of HTs as an alternative and/or complementary approach of OA. Although both small and large animals have been used to develop OA models, the studies concerning the anti-inflammatory effects of HT-containing products were mainly performed on small animals, such as mice, rats, guinea pigs, and rabbits, since they are relatively inexpensive and easy to handle [57].

Compared to the studies on humans, where usually the main objectives were represented by the measurements of functional parameters and analyses of systemic biomarkers of inflammation, studies on animal models are mainly focused on the evaluation of local biochemical markers detected of either biopsy or surgical specimens. However, since each study differs in the type of animal model and OA induction protocol, tested product, vehicle, route of administration, and duration, the comparison of results is very complicated. Nevertheless, evidence on changes of mRNA levels, protein expression, and inflammatory biomarkers, as well as scoring systems, following the administration of HT-containing products have to be discussed.

#### 2.2.1. Expression of Chondrogenic Genes

Only a few studies on animal models [60,63,69] have investigated the effects that the administration of HTs and HT-derived metabolites exerted on genes involved in the chondrogenesis, although it is well-known that the OA process induces chondrocyte dedifferentiation via increased synthesis of type I collagen, and downregulation of the main genes and transcription factors [71]. SRY-Box transcription factor 9 (*Sox9*) is considered one of the master genes which regulate the chondrocyte differentiation process. Since *Sox9* expression stimulates chondrogenesis through the transcriptional activity of entire genes involved in chondrocyte function–e.g., genes encoding for type II collagen (COL2) and aggrecan (ACAN)–the loss of *Sox9* function completely blocks this process. In all studies listed in Table 3, the treatment with HTs or HT-derived metabolites, independently of administration and OA induction protocols, promoted chondrogenesis, upregulating mRNA levels of the abovementioned genes.

Since inflammatory mediators, such as TNF-α and interleukins (e.g., IL-1β, IL-6) stimulate the production of MMP enzymes responsible for the degradation of all components of the ECM, some authors have also investigated the effect of HTs on the *Mmp* mRNA levels. As reported by Mehana et al. (2019) [72], MMP-1 and MMP-13 have predominant roles in OA because they are a rate-limiting factor in the process of collagen degradation. Moreover, the expression of MMP-encoding genes is elevated in arthritis, which is involved in the degradation of ACAN and non-collagen matrix components of cartilage.

Shivnath et al. (2021) [63] have demonstrated a reduction of *Mmp-3* mRNA level associated with the downregulation of the *Cox-2*, which are important mediators of inflammation and key regulators of the OA pathogenesis, following the oral administration of 250 or 500 mg/kg b.w. per day of a pomegranate peel extract in an OA rat model. On the other hand, Yang et al. (2021) [60] and Akhtar et al. (2017) [69] have also shown a significant decrease in *Mmp-9* and *Mmp-13* mRNA levels with the administration of punicalin–a pomegranate ET–and a pomegranate fruit extract, respectively, in two different OA animal models. Moreover, Yang et al. (2021) [60] have investigated the effects of punicalin (100 mg/kg b.w., twice a week for four weeks) in maintaining the normal phenotype of chondrocyte via mediating multiple gene expression in osteoarthritic mice. Punicalin administration resulted in a down-regulation of pivotal genes responsible for the pathogenesis of OA and chondrocyte differentiation, such as runt-related transcription factor 2 (*Runx2*), *Col10a1*, Indian Hedgehog signaling molecule (*Ihh*), and parathyroid hormone-like hormone (*Pthlh*) [60].

It has been established that autophagy is a protective mechanism in normal cartilage, and the expression of autophagy-related genes–e.g., forkhead box O (FOXO) transcription factor –is reduced in OA [73]. Interestingly, punicalin upregulated the mRNA level of *Foxo3* [60]–one of the four members of the *Foxo* family–showing a protective effect on osteoarthritic chondrocytes.

#### 2.2.2. Protein Expression and Inflammatory Biomarkers Level

Up to the present, the discovery of OA-related biomarkers has focused on cartilage, synovial fluid, and serum [74]. Proteomics, immunoassays, and staining strategies have identified many proteins which may relate to pathological mechanisms of OA, such as MMPs, cytokines, collagen and non-collagen proteins, mediators of inflammation, and more recently, autophagy-related proteins.

MMP-13 was analyzed from rat cartilage tissue, showing a significant decrease when punicalagin (PUNI)–a major ET in pomegranate–were orally administered at 10 mg/kg b.w. per day for 12 weeks to the treatment group [65]. Similarly, the levels of the inflammatory cytokines responsible for the MMP synthesis and consequent ECM degradation were significantly reduced both in plasma and synovial fluid by a pomegranate fruit extract (34 mg/kg b.w. daily per eight weeks) orally administered via drinking water in an OA rabbit model [69]. Concerning COL2 and ACAN, only Liu et al. (2021) [65] have analyzed these proteins by western blot, reporting an increase in their expression after the treatment with PUNI. However, Shivnath et al. (2021) [63] have quantified the collagen and glycosaminoglycan content in knee joints of an OA rat model, observing inhibition of collagen degradation and significant retention of the polysaccharide content in the pomegranate peel extract treatment groups (250 or 500 mg/kg b.w. per day for one month).

Liu et al. (2021) [65] have investigated the role of a disintegrin and metalloproteinase with thrombospondin type 5 (ADAMTS5) in the OA pathogenesis since it is responsible for mediating the ACAN degradation in the early stage of the OA. Specifically, they have shown that PUNI was capable of inhibiting ADAMTS5 protein expression and, therefore, the early joint destruction in rats.

COX is a type of oxidoreductase enzyme that plays a key role in the formation of prostaglandins from the arachidonic acid. Since COX-2 expression is upregulated in arthritic synovial membranes and cartilage, and osteoarthritic cartilages release prostaglandin E₂ (PGE_2_)–an eicosanoid modulating cartilage proteoglycan degradation–in levels at least 50-fold higher than normal cartilage [75], inhibition of COX-2 may result in an amelioration of OA. Shukla et al. (2008) [76], in a healthy animal model (non-OA rabbit model), have shown that the administration by gavage of pomegranate fruit extract (34 mg/kg b.w.) significantly inhibited the activity of both COX-1 and COX-2 enzymes, although the inhibitory effect was targeted more towards COX-2. Moreover, as reported by Akhtar et al. (2017) [69], the ad libitum administration of pomegranate fruit extract via drinking water (34 mg/kg b.w. per day for eight weeks) significantly decreased the PGE_2_ level in the synovial fluid of rabbits.

Interestingly, researchers have found that the amount of FOXO proteins is significantly reduced in the joints of humans and mice affected by OA [77]. Recently, Yang et al. (2021) [60] have investigated whether FOXO3 was a target of punicalin to prevent chondrocyte degeneration in vivo. Results have shown that punicalin blocks IL-1β- and TNF-α-triggered phosphorylation and cytoplasmic transfer of FOXO3 protein in OA mice model. Therefore, together with the abovementioned results on the mRNA levels, punicalin might be a potential FOXO3 agonist. On the other hand, Liu et al. (2021) [65] have detected in rat cartilage tissue sections and chondrocytes the levels of FOXO1, proteoglycan 4 (PRG4), hypoxia-inducible factor-3α (HIF3α), ACAN, COL2, phosphor-unc-51 like autophagy activating kinase 1 (p-ULK1), p-Beclin1, microtubule-associated protein light chain 3 (LC3) II/I ratio, ADAMTS5, MMP13, and p62 were analyzed via immunohistochemistry or western blot. The expression of all proteins, except for the last three, increased, suggesting that PUNI promoted autophagy and attenuated ECM degradation in the used rat OA model through FOXO1/PRG4/HIF3α pathway regulation. 

Among HT-derived metabolites administrated to different OA animal models, the effects of urolithin A (Uro-A) on OA symptomatology were evaluated only in the study by Fu et al. (2019) [62], where Uro-A was found to suppress the activation of PI3K/AKT/NF-κB pathways. Therefore, since previous studies [78,79] have shown that the activation of NF-κB can be inhibited by suppressing the PI3K/AKT signaling pathway, which was proved to be therapeutically effective in OA, Uro-A may attenuate the progression of OA.

#### 2.2.3. OA Scoring Systems

Pain scores, histopathology assessments, and functional outcome measures are extensively used in OA animal model studies to evaluate the relative efficacy of therapeutic interventions potentially relevant to human clinical care.

Chronic pain and discomfort are the hallmarks of OA, but their evaluation is highly complex due to the inherent variability associated with the experimental protocol and interpretation of the results. Although Osteoarthritis Research Society International (OARSI) has attempted to uniform OA pain measurements, to date, no such standards exist for the study of chronic pain [57]. Otherwise, OARSI has performed several investigations aimed at providing standard scoring systems for OA histopathological evaluation in mouse [80], rat [81], guinea pig [82], and rabbit [83] models. Based on these scores, several authors [59,61,62,65,66,69] have shown that the administration of HTs in OA animal models reduced synovial thickening and hypercellularity, cartilage degeneration, and ECM loss.

The Mankin score was developed more than 40 years ago, and although it was extensively used in animal models to study OA, it is poorly reproducible and presents challenges while investigating early or intermittent stages of OA. Nevertheless, Shivnath et al. (2021) [63] and Wen et al. (2015) [70] have used this histologic grading system to assess the chondrocyte-protective effects after pomegranate and GA administration in different OA animal models, respectively. Moreover, although the used scores were not specified in the text, also Hadipour-Jahromy and Mozaffari-Kermani (2010) [58] and Tanideh et al. (2016) [68] have shown similar histopathological results on mouse, rat, and guinea pig OA models, respectively.

Concerning functional outcomes, few authors have focused their investigations on the evaluation of parameters related to physical activity, such as weight-bearing, movement, or body weight. Among these authors, Lee et al. (2018) [64] have analyzed the changes in the weight-bearing ratio in an OA-induced rat model after the oral administration of pomegranate peel extract for 28 days. Data have suggested that PUNI, the major compound contained in the extract, alleviated the inflammatory and nociceptive status related to the cartilage breakdown. On the other hand, Shruthi et al. (2014) [67] have shown that the intra-articular injection of EA for 20 days in a formaldehyde-induced OA rat model ameliorated their movement ability and body weight and reduced the paw edema volume. Therefore, they have concluded that EA administration favored the maintenance of synovial membrane and vascular permeability, thereby inhibiting cytokines and leukotriene infiltration, protecting the synovial membrane, and improving general health status.

Hence, among animal studies, it is difficult to understand if the effects achieved with different tested HTs can be effectively translated to human clinical outcomes due to the lack of uniformity in the (i) methodology used to induce OA, (ii) biomarkers evaluated both with protein and gene expression analyses and (ii) measurements of physical activity and histopathological scoring. 

### 2.3. In Vitro and Ex Vivo Studies

In vitro and ex vivo models of OA are widely used to investigate the causes of the disease and the subsequent design and testing of potential therapeutics. However, since a plethora of in vitro/ex vivo models have been implemented and used by researchers through the years (Table 4), it is difficult to establish which are the most appropriate to evaluate the biological effects of HTs.

Models of OA based on the stimulus imparted by cytokines or chemokines, such as IL-1β and TNF-α, are very common since they are reliable and lead to a reproducible inflammatory response. Upon to IL-1β stimulus, chondrocytes produce several inflammatory molecules, such as prostaglandins, COXs, and cytokines. Ding et al. (2020) [84] have demonstrated that Uro-A pre-treatment (up to 15 μM) attenuated IL-1β-induced inflammatory response and cartilage degradation via inhibiting the MAPK cascades in a dose-dependent manner [88] and NF-κB signaling pathway in rat articular chondrocytes. Therefore, Uro-A also reduced the release of inflammatory mediators, such as MMP-3, MMP-9, MMP-13, ADAMTS4, COX-2, and iNOS, and contributed to restoring cartilage ECM, as demonstrated by the increase of COL2 and SOX9 proteins and mRNA levels in vitro, and COL2 and ACAN protein expression on ex vivo cartilage sections [84]. 

Previously, other authors also reported the repression of the activation of MAPK and NF-κB signaling pathways testing pomegranate fruit extracts in primary chondrocytes [85,86,87] or in articular cartilage slides procured from discarded human tissues [85]. However, conclusions that can be drawn from these data are limited due to the use of a whole fruit extract put directly in contact with cells and tissues, not considering the in vivo HT biotransformation.

Previous studies have shown that the activation of NF-κB can also be inhibited by repressing the PI3K/AKT signaling pathway [78]. Fu et al. (2019) [62] have demonstrated that Uro-A significantly inhibited IL-1β-induced PI3K/AKT/NF-κB pathway activation attenuating inflammation and catabolism in human OA chondrocytes both in vitro and in vivo. In in vitro cultured chondrocytes, the pre-treatment with 10 or 30 μM Uro-A for 24 h inhibited the IKBα phosphorylation and degradation, avoiding the subsequent translocation of p65 into the nucleus. Therefore, Uro-A treatment significantly reduced, in a dose-dependent manner, the *iNOS*, *COX-2*, *IL-6*, and *TNF-α* mRNA levels, and the expression of PGE_2_, NO, iNOS, COX-2, TNF-α, IL-6, ADAMTS5, and MMP-13 proteins. Otherwise, the inhibition of the PI3K/AKT/NF-κB pathway has increased the expression of COL2 and ACAN. 

More recently, Lin et al. (2020) [61] have reported the anti-inflammatory effects of EA in both in vitro and in vivo experiments (see Table 3 and Table 4). They have revealed that EA (12.5, 25, and 50 μM) suppressed NF-κB signaling in IL-1β-induced OA in human chondrocytes. Therefore, in agreement with the abovementioned study with Uro-A [84], the inhibition of this pathway caused the reduction of NO, iNOS, COX-2, PGE_2_, IL-6, TNF-α, ADAMTS5, and MMP-13 production. In contrast, EA treatment upregulated the COL2 and ACAN protein expression. On the other hand, some authors [64,76] have directly analyzed the downstream reduction in inflammatory mediators, such as NO, iNOS, COX-2, PGE_2_, and MMP-13. Interestingly, Shukla et al. (2008) [76] have shown a decrease in NO and PGE_2_ production in IL-1β-induced OA in rabbit chondrocytes, which were pre-treated with blood plasma samples procured from overnight food-starved rabbits watered on the day after with 10 mL of pomegranate fruit extract (34 mg/kg b.w.).

Advanced glycation end products (AGEs) are compounds generated through a nonenzymatic reaction between reducing sugars and other macromolecules (proteins, lipids, or nucleic acids), which play an important role in the pathogenesis of OA [89]. Wen et al. (2015) [70] have demonstrated that GA played a chondroprotective role against AGE-induced OA progression. Specifically, they have reported that GA (10–80 μg/mL) had scavenged the ROS by increasing superoxide dismutase (SOD) activity and glutathione content in a dose-dependent manner. Moreover, GA treatment has increased the expression of COL2 and ACAN, as well as prevented the inflammation signaling pathway by reducing the production of NO, iNOS, COX-2, and PGE_2_.

Recently, some authors [59,60,65] have investigated the effects of HTs on the modulation of autophagy in cultured chondrocytes, highlighting whether these molecules may become suitable agents for limiting the progression of cartilage degeneration. However, it is worth noting that the native form of HTs (PUNI [59,65] or punicalin [60]) has been supplemented to cells, thus applying non-physiological conditions, limiting the strength of their results. Nevertheless, the effect of HTs on autophagy had been proven by the same authors by using animal models [59,60,65].

**Figure 1:**
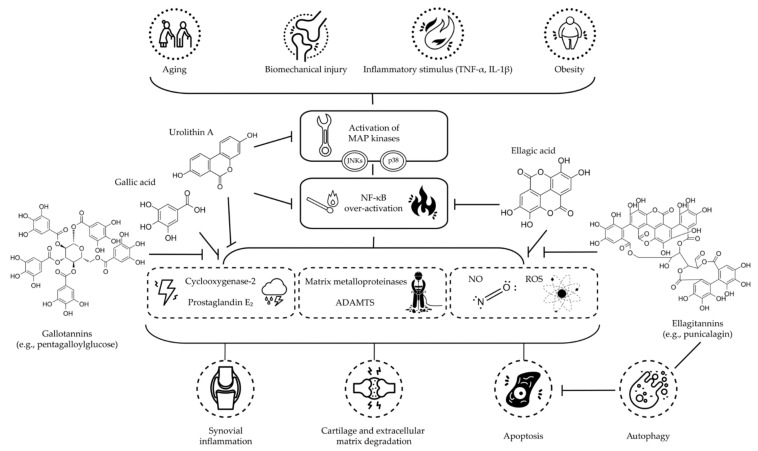
Schematic overview of the proposed mechanisms of action of main dietary HTs and their derived metabolites, discussed in this literature review, on OA (Abbreviations: ADAMTS: short for a disintegrin and metalloproteinase with thrombospondin motifs; IL-1β: interleukin 1β; JNK: c-Jun N-terminal kinase; MAP: mitogen-activated protein; NF-κB: nuclear factor κ light-chain-enhancer of activated B cells; NO: nitric oxide; ROS: reactive oxygen species; TNF-α: tumor necrosis factor α).

Lastly, as for the animal studies, the heterogeneity among the in vitro/ex vivo studies, as well as the supra-physiological loads of cytokines or other inflammatory molecules used to rapidly induce an OA-like pathology into the cells or tissue assessed, make it hard to directly compare the outcomes published in the scientific literature. Hence, based on the here reviewed studies, it can be concluded that HTs and their derived metabolites are capable of inhibiting the activation of MAPK pathways and the over-activation of NF-κB, blocking the consequent signaling cascades and then reducing the synthesis of pro-inflammatory and -apoptotic molecules involved in OA course, also via autophagy modulation (see overview in Figure 1).

## 3. Materials and Methods

### 3.1. Data Sources and Search Strategy

The scientific literature selection process was conducted following PRISMA (Preferred Reporting Items for Systematic Reviews and Meta-Analyses) Extension for Scoping Reviews (PRISMA-ScR) [90]. To identify potentially relevant documents, we performed an extensive search using the MEDLINE and Scopus databases, with no restriction on date of publication, in August 2021; a search update was conducted at the end of December 2021. The following Medical Subject Headings (MeSH) terms and keywords were combined: “pomegranate” or “Punica granatum” or “ellagitannins” or “gallotannins” or “urolithins” and “osteoarthritis”. We did not use any date or language restrictions, and reviews were excluded. The search strategies were as follows: MEDLINE search strategy ((“pomegranate*”[Title/Abstract] OR “Punica granatum”[Title/Abstract] OR “polyphenol*”[Title/Abstract] OR “(poly)phenol*”[Title/Abstract] OR “ellagitannin*”[Title/Abstract] OR “gallotannin*”[Title/Abstract] OR “urolithin*”[Title/Abstract]) AND “osteoarthritis”[Title/Abstract]) NOT “review”[Publication Type]; Scopus search strategy (TITLE-ABS-KEY (pomegranate*) OR TITLE-ABS-KEY (punica AND granatum) OR TITLE-ABS-KEY (polyphenol*) OR TITLE-ABS-KEY ((poly)phenol*) OR TITLE-ABS-KEY (ellagitannin*) OR TITLE-ABS-KEY (gallotannin*) OR TITLE-ABS-KEY (urolithin*) AND TITLE-ABS-KEY (osteoarthritis)) AND (EXCLUDE (DOCTYPE, “re”)). Both search results were exported into EndNote 20.1 (Clarivate, London, UK), and duplicates were removed.

### 3.2. Eligibility Criteria

This review is focused on the possible beneficial effects of dietary high-molecular-weight (poly)phenols (hydrolyzable tannins) against OA based on research studies; therefore, reviews were excluded. Studies using formulations including mixed compounds, other than HTs or their derived metabolites, and those investigating the effects on rheumatoid arthritis were excluded.

### 3.3. Study Selection and Data Extraction

**Figure 2:**
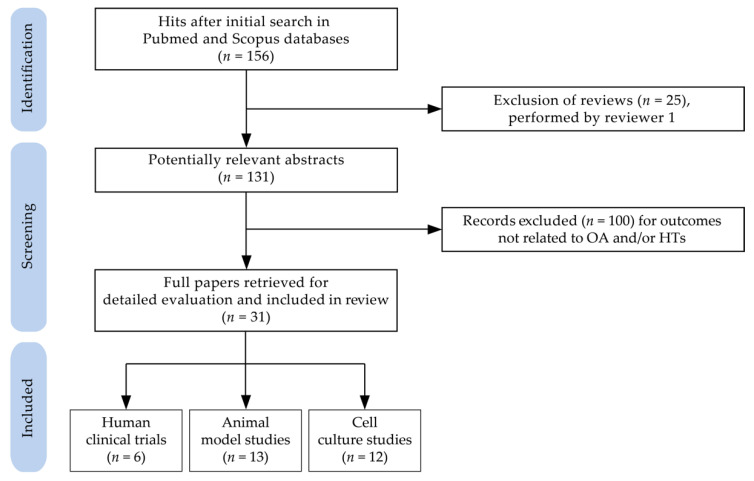
Flow chart of papers included in the review (Abbreviations: HTs: hydrolyzable tannins; OA: osteoarthritis).

Two reviewers–MG and FD–independently selected and evaluated the retrieved articles for eligibility. They sequentially evaluated the title and abstract and then the full text of all potentially relevant publications retrieved by the systematic search. Any disagreements were solved by consensus. All pertinent data to in vivo and in vitro studies were extracted at the level of detail reported in Table 1, Table 3 and Table 4. The flow chart of the selection procedure of the papers is represented in Figure 2.

## 4. Conclusions

OA is a significant cause of welfare and economic morbidity since the number of patients affected by this degenerative disease is continuously increasing due to aging and prolonged human lifespan. However, despite the existence of a wide variety of therapeutic options used for the treatment of pain, there is currently no strategy to restore the chondrocyte phenotype due to the poor intrinsic healing capacity of articular cartilage. Indeed, since OA continues to be one of the main causes of medical consultation [91], the development of effective disease-modifying OA treatments is urgently required, especially because in the most advanced stages of OA, patients require an arthroplasty intervention to replace the joint with a prosthesis.

Although OA may involve an array of diverse external factors acting as triggers (see Figure 1), the overall lack of a standardized approach can limit the comparability of data on their prevention or mitigation associated with consumption or treatment with HTs and HT-containing products, highlighting the need for harmonization to produce reliable results. Nevertheless, based on the here reviewed studies, the association between intake or treatment with HTs and retardation of OA progression has a solid theoretical basis. However, as mentioned above, each class of study shows several limitations and/or shortcomings.

It has become clear that large (poly)phenolic molecules, such as HTs, are broken down by gut microflora and their derivatives are variable metabolized by intestinal and hepatic enzyme systems of a different extent across the human population [40]. HTs have been described to interact with the cytochrome P450 enzymes, and these interactions can affect their bioactivity [92]. Nevertheless, some animal and in vitro studies have not considered the bioavailability and biotransformation of HTs (e.g., treatment with the native form of HTs in biological fluids or culture medium of cells) when evaluating their biological effects (Table 5).

In addition to the aforementioned limitation, cultured cells are often supplemented with unphysiological, high concentrations of compounds that, in vivo, cannot be achieved in the cellular milieu and, in a closed system, can have paradoxical effects. For instance, PUNI and pomegranate extract can act as a prooxidant rather than an antioxidant when supplemented into cell culture media at very high doses [93]. The de facto application of relatively high concentrations of (poly)phenols in their native form to cultured cells remains a major limitation of in vitro research that is still published nowadays, as previously underlined by Aragonès et al. (2017) [94] and Ávila-Gálvez et al. (2018) [95]. Further studies need to overcome these critical limitations to assert the real bioactive metabolites and the potential mechanisms responsible for the efficacy of HTs on OA, paving the way towards the development of alternative strategies for the improvement of pro-inflammatory cytokine-induced chondrocyte dysfunctions and injuries.

## Figures and Tables

**Table 1:** Summary of findings related to HT-containing product consumption and OA in humans (see Table S1 for specific details on sample preparation and characterization of the tested products).

Study Design	Population	Subjects (Gender, No., Age, BMI, Physical Activity)	Intervention	Control/Comparator	Duration	Main OA-Related Outcomes	Reference
Randomized, pre- and post-test, parallel-arm	Adults with knee OA	F: 34, M: 4; 41–66 years; BMI: 23–36; I: 17, LA: 17, A: 4	Pomegranate juice 200 mL/day	–	6 weeks	Clinical indexes: ↔ WOMAC pain, ↓ WOMAC stiffness, physical function, and total score. Biochemical outcomes: ↔ serum MMP-1, MMP-13 and ↑ GPx serum levels	Ghoochani et al. 2016 [43]
Randomized, double-blind, placebo-controlled, parallel-arm	Adult obese women with knee OA	F: 60; 40–58 years; BMI: 30–34; I: 0, LA: 46, A: 14	Encapsulated pomegranate peel hydroalcoholic extract 500 mg b.i.d.	Placebo 1 capsule b.i.d.	8 weeks	Biochemical outcomes: ↑ KOOS, ↓ VAS	Rafraf et al. 2017 [44]
Randomized, double-blind, placebo-controlled, parallel-arm	Adult obese women with knee OA	F: 60; 40–58 years; BMI: 30–34; I: 0, LA: 46, A: 14	Encapsulated pomegranate peel hydroalcoholic extract 500 mg b.i.d.	Placebo 1 capsule b.i.d.	8 weeks	Biochemical outcomes: ↓ MDA, ↑ SOD, GPx, and TAC activity	Haghighian et al. 2021 [45]
Randomized, double-blind, placebo-controlled, crossover	Obese adults with knee OA	F: 13, M: 4; 50–64 years; BMI: 38–41	Freeze-dried strawberry powder 50 g b.i.d. (equivalent to ≈ 500 g of fresh strawberries)	Placebo powder 50 g b.i.d.	12 weeks	Clinical indexes: ↓ HAQ-DI, ↔ pain VAS, ↓ ICOAP constant pain, intermittent pain, and total pain. Biochemical outcomes: ↔ CRP, MMP-8, and nitrite levels, ↓ IL-1β, IL-6, and MMP-3 levels	Schell et al. 2017 [46]
Randomized, double-blind, placebo-controlled, crossover	Obese adults with knee OA	F: 13, M: 4; 54–60 years; BMI: 38–41	Freeze-dried strawberry powder 50 g b.i.d. (equivalent to ≈ 500 g of fresh strawberries)	Placebo powder 50 g b.i.d.	12 weeks	Biochemical outcomes: ↓ TNF-α, TNF-R2, 4-HNE, and CD levels, ↔ IL-19, CD163, TNF-R1, and PTX3 levels	Basu et al. 2018 [47]
Randomized, double-blind placebo-controlled, parallel-arm	Obese adults with knee OA	F: 47, M: 16; 55–57years; BMI: 31–33	Freeze-dried blueberry powder 40 g/day	Placebo powder 40 g/day	4 months	Clinical indexes: ↓ WOMAC pain, stiffness, physical function, and total score. Biochemical outcomes: ↔ TNF-α, IL-1, IL-6, IL-10, IL-13, MMP-3, MMP-13, and MCP-1 levels	Du et al. 2019 [48]

Abbreviations: ↑: increase; ↔: no change; ↓: decrease; 4-HNE: 4-hydroxy-2-nonenal; A: active; ADL: activities of daily living (ADL) questionnaires; b.i.d.: bis in die (twice a day); BMI: body mass index; CAP-e: cell-based antioxidant protection in erythrocytes; CD: conjugated dienes; CD163: cluster of differentiation 163; CRP: C-reactive protein; F: female; GPx: glutathione peroxidase; HAQ-DI: health assessment questionnaire disability index; hsCRP: high-sensitivity C-reactive protein; I: inactive; ICOAP: intermittent and constant osteoarthritis pain; IL: interleukin; KOOS: knee injury and osteoarthritis outcome score; LA: low active; M: male; MCP: monocyte chemoattractant protein; MDA: malondialdehyde; MMP: matrix metalloproteinase; OA: osteoarthritis; oz: ounce; PTX3: pentraxin 3; ROM: range of motion; SOD: superoxide dismutase; TAC: total antioxidant capacity; TNF-R: tumor necrosis factor receptor; TNF-α: tumor necrosis factor α; VAS: visual analogue scale; WOMAC: Western Ontario and McMaster Universities Osteoarthritis Index.

**Table 2:** Registered clinical trials testing the effects of HT-containing foodstuffs and products. Source: Cochrane Central Register of Controlled Trials (CENTRAL) [52].

CENTRAL Identifier	Study Focus	Intervention/ Treatment	Study Design, Duration	Sponsor	Estimated Enrolment	Study Start Date	Completion Date	Reference
IRCT2014031517017N1	Knee OA	Pomegranate juice	Double-blind, placebo-controlled, parallel-arm, randomized, 6 weeks	Ahvaz Jundishapur University of Medical Sciences (Ahvaz, Iran)	50	March 2014	March 2015	[54]
IRCT201405183664N11	Knee OA	Pomegranate pericarp hydroalcoholic extract	Double-blind, placebo-controlled, parallel-arm, randomized, 2 months	Tabriz University of Medical Sciences (Tabriz, Iran)	30	August 2014	December 2014	[55]
NCT02518347	Knee OA	Freeze-dried strawberries	Triple-blind, placebo-controlled, crossover, randomized, 12 weeks	Oklahoma State University (Stillwater, OK, USA)	20	March 2015	May 2017	[56]
NCT03703024	Knee OA	Raspberry leaf extract	Double-blind, placebo-controlled, parallel-arm, randomized, 12–13 weeks	Atlantia Food Clinical Trials (Blackpool, Munster, Ireland)	195	June 2017	December 2018	[53]

**Table 3:** Overview of the effects of HT-containing products in OA animal models (see Table S1 for specific details on sample preparation and bioactive compound content in the tested products).

Species	Animal Model Characteristics (Sex, Age, Weight)	Tested Product(s), Vehicle, Route of Administration, Duration	OA Induction	Effects	Reference
Mouse	BALB/c, male, 20–25 g	Pomegranate juice (4, 10, 20 mL/kg b.w. b.i.d. in divided doses) via oral gavage for 2 weeks	MIA intra-articular injection	↓ MIA effects (especially with the 20 mL/kg dose)	Hadipour-Jahromy and Mozaffari-Kermani 2010 [58]
	C57BL/6, male, 7–8-week-old, 20–25 g	PUNI (20 mg/kg b.w. per day) via oral gavage for 8 weeks	DMM	↓ cellular apoptosis, ↓ OARSI score	Kong et al. 2020 [59]
	C57BL/6J, male, 10-week-old	Punicalin (100 mg/kg b.w. per day, twice a week) in PBS via gastric gavage for 4 weeks	10 ng IL-1β and 50 ng TNF-α in 5-μL PBS (twice a week for 4 weeks)	↓ FOXO3 phosphorylation, ↑ *Sox9*, *Col2a1*, and *Foxo3* gene expression, ↓ *Mmp-9*, *Mmp-13*, *Col10a1*, *Runx2*, *Ihh*, and *Pthlh* gene expression	Yang et al. 2021 [60]
	C57BL/6 wild type, male, 10-week-old	EA (40 mg/kg b.w. every 2 days) in saline via gastric gavage for 8 weeks	DMM	↓ OARSI score, ↓ synovitis score	Lin et al. 2020 [61]
	C57BL/6 wild type, male, 1-week-old	Uro-A (20 mg/kg b.w. per day) in CMC-Na (0.5%) via gastric gavage for 8 weeks	DMM	↓ OARSI scores, ↓ p-PI3K and p-AKT-positive chondrocytes, ↓ p65-positive nuclei	Fu et al. 2019 [62]
Rat	Sprague Dawley, female, 100–150 g	Pomegranate peel hydroalcoholic extract (250 or 500 mg/kg b.w. per day) via oral administration for 1 month	Collagenase II	↓ Mankin score, ↓ serum ALP, ↓ *Mmp-3* and *Cox-2* gene expression, ↑ *Col2* gene expression, ↑ collagen and glycosaminoglycan content	Shivnath et al. 2021 [63]
	Wistar, ≈300 g	Pomegranate peel acetone extract (15 and 150 mg/kg b.w.) via oral administration for 28 days	Collagenase II	↓ weight-bearing ratio	Lee et al. 2018 [64]
	Sprague Dawley, male, 8-week-old, 300–350 g	PUNI (10 mg/kg b.w. per day) in saline via oral gavage for 12 weeks	ACLT-MCLT-DMM	↓ OARSI score, ↓ apoptosis rate, ↑ protein expression of FOXO1, PRG4, HIF3α, ACAN, COL2, p-ULK1, p-Beclin1, LC3II/I ratio, ↓ protein expression of ADAMTS5, MMP-13, and p62	Liu et al. 2021 [65]
	Sprague-Dawley, male, 200–250 g	PUNI (30 μL of 9.2 mM, twice a week, in saline, via intravenous injection for 5 weeks	MIA intra-articular injection	↓ OARSI cartilage matrix width loss measurements	Elder et al. 2021 [66]
	Wistar albino rats, male, 150–200 g	EA (250 mg/mL) in saline via intra-articular injection for 20 days	Formaldehyde	↓ paw edema volume, ↑ movement ability, ↑ b.w.	Shruthi et al. 2014 [67]
Guinea pig	Short-haired England, male, 700–800 g	Mango fruit hydroalcoholic extract (500 mg/kg b.w. per day) via oral gavage for 8 weeks (oral treatment) or intra-articular injection 2 times with a time interval of 4 weeks (injection treatment)	ACLT	↑ radiological and histopathological assessments (intra-articular injection only)	Tanideh et al. 2016 [68]
Rabbit	New Zealand White rabbits, male, 8-month-old	Pomegranate fruit hydroalcoholic extract (34 mg/kg b.w. per day) via drinking water, *ad libitum*, for 8 weeks	ACLT	↓ OARSI score, ↓ *Mmp-3*, *Mmp-9*, and *Mmp-13* gene expression, ↑ *Acan* and *Col2a1* gene expression, ↓ IL-1β, IL-6, and PGE_2_ levels in plasma and synovial fluid	Akhtar et al. 2017 [69]
	New Zealand rabbits, male, 3-week-old, 400–600 g	GA (0.5 mL of 80 μM) in PBS via intra-articular injection every 72 h for 8 consecutive weeks	Collagenase II	↓ Mankin score	Wen et al. 2015 [70]

Abbreviations: ↑: increase; ↔: no change; ↓: decrease; ACAN: aggrecan; ACLT: anterior cruciate ligament transection; ADAMTS5: A disintegrin and metallopeptidase with thrombospondin type 1 Motif 5; ALP: alkaline phosphatase; b.i.d.: bis in die (twice a day); b.w.: body weight; CMC-Na: sodium carboxymethyl cellulose; COL2: collagen type II; COL2A1: collagen type II α 1; COL10A1: collagen type X α 1; COX: cyclooxygenase; DMM: destabilization of the medial meniscus; EA: ellagic acid; FOXO: forkhead box O; GA: gallic acid; HIF3α: hypoxia inducible factor 3 subunit α; Ihh: Indian Hedgehog signaling molecule; IL: interleukin; LC3: microtubule-associated protein light chain 3; MCLT: medial collateral ligament transection; MIA: monosodium iodoacetate; MMP: matrix metalloproteinase; OARSI: Osteoarthritis Research Society International; p-AKT: phospho-serine/threonine kinase; PBS: phosphate buffered saline; p-PI3K: phosphor-phosphoinositide 3-kinase; p-ULK: phospho-unc-51 like autophagy activating kinase; p65: p 65 protein (nuclear factor NF-kappa-B p65 subunit); PGE_2_: prostaglandin E2; PRG4: proteoglycan 4; Pthlh: parathyroid hormone like hormone; PUNI: punicalagin; RUNX: runt-related transcription factor; SOX9: SRY-Box transcription factor 9; TNF-α: tumor necrosis factor α; Uro-A: urolithin A.

**Table 4:** Summary of the biological effects of HTs or HT-derived metabolites assayed in cell culture studies (see Table S1 for specific details on sample preparation and characterization of the tested compounds).

Cell Model	Primary Cell/Cell Line/Tissue	Tested Compound(s), Dose, Duration	Pro-Inflammatory Treatment	Biological Effects	Reference
Mouse chondrocytes	Primary chondrocytes from immature C57BL/6 mice	PUNI (25 or 50 μg/mL) for 24 h	100 μM TBHP	↑ protein expression of ATG12-5, LC3 II/I, p-ULK, Beclin1, Bcl-2, COL2, HO-1, SOD1, and NQO1, ↓ protein expression of BAX, cleaved caspase 3, ADAMTS5, MMP-3 and MMP-13, ↓ p62 protein expression (dose-dependent)	Kong et al. 2020 [59]
	Primary chondrocytes C57BL/6J mice	Punicalin (80 or 100 μg/mL) for 24 h	10 ng/mL IL-1β and 50 ng/mL TNF-α	↓ p-FOXO3 protein expression, ↑ *Sox9* and *Col2a1* mRNA levels, ↓ *Col10a1*, *Mmp-9*, *Ihh*, *Pthlh*, and *Runx2* mRNA levels, ↔ *Mmp-13*, *Col9a1*, and *Runx3* mRNA levels (dose-dependent)	Yang et al. 2021 [60]
Rat chondrocytes	Primary chondrocytes from Wistar rats	Pomegranate peel acetone extract (12.5–100 μg/mL) or PUNI (25–50 μg/mL) for 16 h as co-treatment with pro-inflammatory stimulus	10 ng/mL IL-1β	↓ iNOS, MMP-13, and COX-2 protein expression, ↓ PGE_2_ release (dose-dependent)	Lee et al. 2018 [64]
	Primary chondrocytes from 5-day-old Sprague Dawley rats	PUNI (25, 50, and 100 μM of) for 8 h prior to pro-inflammatory stimulus	10 ng/mL of LPS	↑ mRNA levels and protein expression of FOXO1, PRG4, HIF3α, p-ULK1, p-Beclin1, and LC3 II/I ratio, ↓ p62 protein expression	Liu et al. 2021 [65]
	Primary chondrocytes from 2- (in vitro study) and 4-week-old (ex vivo study) Sprague Dawley rats	Uro-A (1–15 μM) for 2 or 3 days, or 2 h prior to pro-inflammatory stimulus	20 ng/mL IL-1β for 2 days (in vitro) or 30 ng/mL IL-1β for 3 days (ex vivo)	in vitro: ↓ protein expression of MMP-3, MMP-9, MMP-13, ADAMTS4, COX-2 and iNOS, ↑ gene and protein expression of COL2 and SOX9, ↓ p-NF-κB, p65, p-ERK1/2, p-JNK, and p-p38 MAPK protein expression (dose-dependent); ex vivo: ↓ OARSI score, ↑ COL2 and ACAN protein expression (dose-dependent)	Ding et al. 2020 [84]
Rabbit chondrocytes	Primary chondrocytes from 1-year-old New Zealand white male rabbits	Blood plasma from rabbits given pomegranate fruit extract (10 mL, 34 mg/kg b.w., via oral gavage for 48 h) for 2 h prior to pro-inflammatory stimulus	5 ng/mL IL-1β for 24	↓ PGE_2_ and NO production	Shukla et al. 2008 [76]
	Primary chondrocytes from 3-week-old New Zealand male rabbits	GA (10–80 μg/mL) for 48 h prior to pro-inflammatory stimulus	100 μg/mL AGEs	↑ *Col2a1* gene expression, ↑ COL2 and ACAN protein expression, ↓ iNOS and COX-2 protein expression, ↓ NO and PGE_2_ release, ↑ SOD activity and GSH content, ↓ ROS release (dose-dependent)	Wen et al. 2015 [70]
Human chondrocytes	Primary chondrocytes (in vitro) and articular cartilage slides (ex vivo)	Pomegranate fruit extract (6.25–50 mg/L) for 24 h (in vitro), pomegranate fruit extract (25 or 50 mg/L) for 72 h (ex vivo)	5 μg/L IL-1β (in vitro), 10 μg/L IL-1β (ex vivo)	in vitro: ↓ MMP-1, MMP-3, MMP-13 gene and protein expression, ↓ protein expression of p-ERK, p-JNK, p-p38-MAPK, c-JUN, ATF-2, NF-κB, and p-IκBα (dose-dependent); ex vivo: ↓ glycosaminoglycan release (dose-dependent)	Ahmed et al. 2005 [85]
	Primary chondrocytes	Pomegranate fruit extract (10 or 50 μg/mL) for 2 h after an overnight serum starvation	10 ng/mL IL-1β	↓ IL-6 gene and protein expression, ↓ ROS level, ↓ NF-κB/p65, ↑ IκBα, ↓ p-IKKα/β, ↓ p-IKKβ gene and protein expression (dose-dependent)	Haseeb et al. 2017 [86]
	Primary chondrocytes from 19 patients (58- 77 years, 14 women and 5 men)	Pomegranate fruit extract (6.25 to 100 μg/mL) for 1 or 2 h after overnight serum starvation, prior to pro-inflammatory stimulus	10 ng/mL IL-1β	↓ protein expression of p-MKK3, p-MKK6, p-p38 MAPKα, and RUNX2 (dose-dependent)	Rasheed et al. 2010 [87]
	Primary chondrocytes from 8 patients (52–73 years, 4 women and 4 men)	EA (12.5, 25, and 50 μM) for 24 or 48 h prior to pro-inflammatory stimulus	10 ng/mL IL-1β	↓ NO, iNOS, PGE_2_, COX-2, IL-6, TNF-α, ADAMTS5, and MMP-13 levels, ↓ *iNOS* and *COX-2* gene expression, ↑ COL2 and ACAN protein expression, ↓ NF-κB protein expression (dose-dependent)	Lin et al. 2020 [61]
	Primary chondrocytes from 6 patients (65–70 years, 3 women and 3 men)	Uro-A (3, 10, and 30 μM) for 24 h prior to pro-inflammatory stimulus	10 ng/mL IL-1β	↓ *iNOS*, *COX-2*, *IL-6*, and *TNF-α* mRNA levels, ↓ PGE_2_, NO, iNOS, TNF-α, IL-6, COX-2, ADAMTS5, and MMP-13 levels, ↑ COL2 and ACAN protein expression, ↑ IκBα, ↓ p65, ↓ p-PI3K, and p-AKT protein expression (dose-dependent)	Fu et al. 2019 [62]

Abbreviations: ↑: increase; ↔: no change; ↓: decrease; ACAN: aggrecan; ADAMTS4: ADAM metallopeptidase with thrombospondin type 1 Motif 4; ADAMTS5: ADAM metallopeptidase with thrombospondin type 1 Motif 5; AGE: advanced glycation end product; ATF-2: activating transcription factor 2; ATG12-5: autophagy-related protein 12-5; b.w.: body weight; BAX: Bcl-2-like protein 4; COL2: collagen type II; COL2A1: collagen type II α 1; COL9A1: collagen type IX α 1; COL10A1: collagen type X α 1; COX: cyclooxygenase; EA: ellagic acid; FOXO: forkhead box O; GA: gallic acid; GSH: glutathione; HO-1: heme oxygenase 1; Ihh: Indian Hedgehog signaling molecule; IKKα/β: I-kappa B Kinase α/β; IL: interleukin; iNOS: inducible nitric oxide synthase; IκB: nuclear factor of kappa light polypeptide gene enhancer in B-cells inhibitor; LPS: lipopolysaccharides; MAPK: mitogen-activated protein kinase; MKK: mitogen activated protein kinase; MMP: matrix metalloproteinase; NF-κB: nuclear factor κ light-chain-enhancer of activated B cells; NO: nitric oxide; NQO1: NAD(P)H quinone dehydrogenase 1; OARSI: Osteoarthritis Research Society International; p-AKT: phospho-serine/threonine kinase; p-ERK: phosphor-extracellular signal-regulated kinase; p-FOXO3: phospho-forkhead box O3; p-IKK: phospho-IKK; p-IκB: phospho-IκB; p-JNK: phosphor-c-Jun N-terminal kinase; p-MKK: phosphated MKK; p-NF-κB: phosphated NF-κB; p-p38 MAPK: phospho-mitogen-activated protein kinase p38; p-PI3K: phosphor-phosphoinositide 3-kinase; p-ULK: phospho-unc-51 like autophagy activating kinase; p65: p 65 protein (nuclear factor NF-kappa-B p65 subunit); PGE_2_: prostaglandin E2; PRG4: proteoglycan 4; Pthlh: parathyroid hormone like hormone; PUNI: punicalagin; ROS: reactive oxygen species; RUNX: runt-related transcription factor; SOD: superoxide dismutase; SOX9: SRY-Box transcription factor 9; TBHP: tert-butyl hydroperoxide; TNF-α: tumor necrosis factor α; Uro-A: urolithin A.

**Table 5:** Published animal model and cell culture studies on the potential beneficial effects on OA of HT-containing products where biotransformation of HTs into derived metabolites has been evaluated or taken into account.

Research Study Type	Studies Where Bioavailability and Metabolism of HTs Have Been Considered ^†^	Studies Where Bioavailability and Metabolism of HTs Have Not Been Considered ^‡^
Animal model studies	Akhtar et al., 2017 [69], Fu et al., 2019 [62], Hadipour-Jahromy and Mozaffari-Kermani 2010 [58], Kong et al., 2020 [59], Lee et al., 2018 [64], Lin et al., 2020 [61], Liu et al., 2021 [65], Shivnath et al., 2021 [63], Shruthi et al., 2014 [67], Tanideh et al., 2016 (oral treatment) [68], Wen et al., 2015 [70], Yang et al. 2021 [60]	Ding et al., 2020 [84], Fu et al., 2019 [62], Lin et al., 2020 [61], Shukla et al., 2008 [76], Wen et al., 2015 [70]
Cell culture studies	Ding et al., 2020 [84], Fu et al., 2019 [62], Lin et al., 2020 [61], Shukla et al., 2008 [76], Wen et al., 2015 [70]	Ahmed et al., 2005 [85], Haseeb et al., 2017 [86], Kong et al., 2020 [59], Lee et al., 2018 [64], Liu et al., 2021 [65], Rasheed et al., 2010 [87], Yang et al., 2021 [60]

^†^ e.g., use of the newly formed metabolites of HTs in biological fluids or culture medium of cells. ^‡^ e.g., use of the native form of HTs in biological fluids or culture medium of cells other than enterocytes.

## Data Availability

Not applicable.

## Supplementary Materials

The following supporting information can be downloaded. Table S1: Detailed description of sample preparation and bioactive compound content in the tested products of the reviewed studies.
