# Supplementary material for: Do Pomegranate Hydrolyzable Tannins and Their Derived Metabolites Provide Relief in Osteoarthritis? Findings from a Scoping Review
Source: Molecules. 2022 Feb 3;27(3):1033. doi: 10.3390/molecules27031033 (PMC8840395; doi:10.3390/molecules27031033)
Supplement: Supplementary file 1 [file molecules-27-01033-s001.zip › molecules-1558207-supplementary.pdf]

SUPPLEMENTARY TABLE

Table S1. Detailed description of sample preparation and bioactive compound content in the tested products of the reviewed studies.

Reference	Tested Product	Content of the Bioactive Compound(s)	Method of Preparation / Supplier
**Human Studies**			
Ghoochani et al. 2016 [1]	Pomegranate juice	n.a.	n.a.
Rafraf et al. 2017 [2]	Pomegranate peel hydroalcoholic extract	90% EA on a dry basis	Pomegranate peels were manually separated after juicing and dried under vacuum at 40°C. Then dried peels were grounded in a mill and then subjected to extraction using ethanol-water 80% by the maceration method. The extract was filtered and dried under vacuum at 50°C.
Haghighian et al. 2021 [3]	Pomegranate peel hydroalcoholic extract	n.a.	See Haghighian et al. 2021 [3]
Schell et al. 2017 [4]	Freeze-dried strawberry powder	TPC: 1585 mg of GAE<br>Total anthocyanins: 66 mg of cyanidin-3-glucoside equivalents<br>EA: 220 mg	California Strawberry Commission (USA)
Basu et al. 2018 [5]	Freeze-dried strawberry powder	TPC: 1585 mg of GAE<br>Total anthocyanins: 66 mg of cyanidin-3-glucoside equivalents<br>EA: 220 mg	California Strawberry Commission (USA)
Du et al. 2019 [6]	Freeze-dried blueberry powder	n.a. (referred to a website no longer available)	US Highbush Blueberry Council (USA)
**Animal Model Studies**			
Hadipour-Jahromy and Mozaffari-Kermani 2010 [7]	Pomegranate juice	n.a.	The fruit seeds were manually separated from the pericarps, and the juice was prepared daily using an electric juice.
Kong et al. 2020 [8]	PUNI	–	Meilun Bio. (China), cat. no: MB6504
Yang et al. 2021 [9]	Punicalin	–	Sigma-Aldrich, cat. no: PHL83532
Lin et al. 2020 [10]	EA	purity ≥ 98%	Solarbio (China)
Fu et al. 2019 [11]	Uro-A	purity ≥ 98%	Herbpurify (China)
Shivnath et al. 2021 [12]	Pomegranate peel hydroalcoholic extract	TPC: 633 ± 1.16 mg of GAE per g D.W.;<br>TFC: 420.3 ± 2.14 mg RE per gram D.W.	The peels of pomegranate were shade dried and subsequently powdered. The 95% ethanolic extract of the powder was prepared using the Soxhlet apparatus. The pooled filtrates were further concentrated under reduced pressure at 40°C.
Lee et al. 2018 [13]	Pomegranate peel acetone extract	Punicalagin: 19.1% (concentration range of punicalagin in the calibration curve 25–400 μg/mL)	The pulverized pomegranate peel was homogenized with 70% aqueous acetone, filtered, concentrated, and further freeze-dried to yield 70% acetone extract of pomegranate peels.

Liu et al. 2021 [14]	PUNI	purity 98%	Feiyubio (Nantong, China)
Elder et al. 2021 [15]	PUNI	purity 98%	Biopurify Phytochemicals Ltd. (Sichuan, China),
Shruthi et al. 2014 [16]	EA	–	The leaves of *Kirganelia reticulata* were shade-dried and then powdered using a mechanical grinder. The powder was subjected to extraction using hexane, chloroform, and methanol separately using Soxhlet apparatus. The extracts were evaporated to dryness using a Rotavapor. The obtained methanolic extract was used for the isolation of EA.
Tanideh et al. 2016 [17]	Mango fruit hydroalcoholic extract	n.a.	The shade dried epicarp and mesocarp parts of the fruits were chopped into small pieces and then crushed into powder by a blender. The powder was macerated in hydroalcoholic solution (50%) and stored in a percolator for 72 h. The extract was dried by a desiccator in 24 h. The extract was then filtered by cellulose acetate membrane syringe filter (pore size 0.2 μm).
Akhtar et al. 2017 [18]	Pomegranate fruit hydroalcoholic extract	n.a.	The extract was prepared by extraction of fruit residue after pressing for juice and solid-phase extraction.
Wen et al. 2015 [19]	GA	–	Sigma-Aldrich (USA)
**Cell Culture Studies**			
Kong et al. 2020 [8]	PUNI	–	Meilun Bio. (China), cat. no: MB6504
Yang et al. 2021 [9]	Punicalin	–	Sigma-Aldrich, cat. no: PHL83532
Lee et al. 2018 [13]	Pomegranate peel acetone extract	Punicalagin: 19.1% (concentration range of punicalagin in the calibration curve 25–400 μg/mL)	The pulverized pomegranate peel was homogenized with 70% aqueous acetone, filtered, concentrated, and further freeze-dried to yield 70% acetone extract of pomegranate peels.
Liu et al. 2021 [14]	PUNI	purity: 98%	Feiyubio (China)
Ding et al. 2020 [20]	Uro-A	–	Cayman Chemical (USA)
Shukla et al. 2008 [21]	Pomegranate fruit extract	TPC: 107.5 ± 3 mg/g TAE	The methanol-soluble fraction was filtrated, condensed, and freeze-dried.
Wen et al. 2015 [19]	GA	–	Sigma-Aldrich (USA)
Ahmed et al. 2005 [22]	Pomegranate fruit extract	n.a.	The methanol-soluble fraction was freeze-dried.
Haseeb et al. 2017 [23]	Pomegranate fruit extract	TPC: 107.5 ± 3 mg/g TAE	Pomegranate fruit extract is produced in a 2-step process: 1) extraction of fruit residue after pressing for juice and 2) solid-phase extraction to produce a powder.
Rasheed et al. 2010 [24]	Pomegranate fruit extract	TPC: 107.5 ± 3 mg/g TAE	See Haseeb et al. 2017 [23]
Lin et al. 2020 [10]	EA	purity ≥ 98%	Solarbio (China)
Fu et al. 2019 [11]	Uro-A	purity ≥ 98%	Herbpurify (China)

Abbreviations: D.W.: dry weight; EA: ellagic acid; GA: gallic acid; GAE: gallic acid equivalent; n.a.: not available; PUNI: punicalagin; RE: rutin equivalent; TAE: tannic acid equivalent; TFC: total flavonoids content; TPC: total phenolic content; Uro-A: urolithin A.

References

1. Ghoochani, N.; Karandish, M.; Mowla, K.; Haghighizadeh, M.H.; Jalali, M.T., The effect of pomegranate juice on clinical signs, matrix metalloproteinases and antioxidant status in patients with knee osteoarthritis. *J Sci Food Agric* **2016**, *96*, 4377-4381.
2. Rafraf, M.; Hemmati, S.; Jafarabadi, M.A.; Moghaddam, A.; Haghighian, M.K., Pomegranate (*Punica Granatum* L.) peel hydroalcoholic extract supplementation reduces pain and improves clinical symptoms of knee osteoarthritis: A randomized double-blind placebo controlled study. *Iran Red Crescent Med J* **2017**, *19*, e38577.
3. Haghighian, M.K.; Rafraf, M.; Hemmati, S.; Haghravan, S.; Asghari-Jafarabadi, M., Effects of pomegranate (*Punica granatum* L.) peel extract supplementation on serum lipid profile and oxidative stress in obese women with knee osteoarthritis: A double blind, randomized, placebo controlled study. *Adv Integr Med* **2021**, *8*, 107-113.
4. Schell, J.; Scofield, R.H.; Barrett, J.R.; Kurien, B.T.; Betts, N.; Lyons, T.J.; Zhao, Y.D.; Basu, A., Strawberries improve pain and inflammation in obese adults with radiographic evidence of knee osteoarthritis. *Nutrients* **2017**, *9*, 949.
5. Basu, A.; Kurien, B.T.; Tran, H.; Byrd, B.; Maher, J.; Schell, J.; Masek, E.; Barrett, J.R.; Lyons, T.J.; Betts, N.M., *et al.*, Strawberries decrease circulating levels of tumor necrosis factor and lipid peroxides in obese adults with knee osteoarthritis. *Food Funct* **2018**, *9*, 6218-6226.
6. Du, C.; Smith, A.; Avalos, M.; South, S.; Crabtree, K.; Wang, W.; Kwon, Y.H.; Vijayagopal, P.; Juma, S., Blueberries improve pain, gait performance, and inflammation in individuals with symptomatic knee osteoarthritis. *Nutrients* **2019**, *11*, 290.
7. Hadipour-Jahromy, M.; Mozaffari-Kermani, R., Chondroprotective effects of pomegranate juice on monoiodoacetate-induced osteoarthritis of the knee joint of mice. *Phytother Res* **2010**, *24*, 182-185.
8. Kong, J.; Wang, J.; Gong, X.; Zheng, X.; Chen, T., Punicalagin inhibits *tert*-butyl hydroperoxide-induced apoptosis and extracellular matrix degradation in chondrocytes by activating autophagy and ameliorates murine osteoarthritis. *Drug Des Devel Ther* **2020**, *14*, 5521-5533.
9. Yang, L.; Fan, C.; Shu, T.; Wang, S., Punicalin alleviates TNF-α- and IL-1β-induced chondrocyte dysfunction and cartilage metabolism via mediating FOXO3 signaling axis. *J Food Biochem* **2021**, e13755.
10. Lin, Z.; Lin, C.; Fu, C.; Lu, H.; Jin, H.; Chen, Q.; Pan, J., The protective effect of ellagic acid (EA) in osteoarthritis: An *in vitro* and *in vivo* study. *Biomed Pharmacother* **2020**, *125*, 109845.
11. Fu, X.; Gong, L.F.; Wu, Y.F.; Lin, Z.; Jiang, B.J.; Wu, L.; Yu, K.H., Urolithin A targets the PI3K/Akt/NF-κB pathways and prevents IL-1β-induced inflammatory response in human osteoarthritis: *in vitro* and *in vivo* studies. *Food Funct* **2019**, *10*, 6135-6146.
12. Shivnath, N.; Rawat, V.; Siddiqui, S.; Verma, S.; Gupta, P.; Rais, J.; Khan, M.S.; Arshad, M., Antiosteoarthritic effect of *Punica granatum* L. peel extract on collagenase induced osteoarthritis rat by modulation of COL-2, MMP-3, and COX-2 expression. *Environ Toxicol* **2021**, *36*, 5-15.
13. Lee, C.J.; Chen, L.G.; Liang, W.L.; Hsieh, M.S.; Wang, C.C., Inhibitory effects of punicalagin from *Punica granatum* against type II collagenase-induced osteoarthritis. *J Funct Foods* **2018**, *41*, 216-222.
14. Liu, F.; Yang, H.; Li, D.; Wu, X.; Han, Q., Punicalagin attenuates osteoarthritis progression via regulating Foxo1/Prg4/HIF3α axis. *Bone* **2021**, *152*, 116070.
15. Elder, S.H.; Mosher, M.L.; Jarquin, P.; Smith, P.; Chironis, A., Effects of short-duration treatment of cartilage with punicalagin and genipin and the implications for treatment of osteoarthritis. *J Biomed Mater Res B Appl Biomater* **2021**, *109*, 818-828.
16. Shruthi, S.D.; Sujan Ganapathy, P.S.; Kumar, R.; Shivakumara; Dharshan, J.C.; Ramachandra, Y.L., *In vivo*, *in vitro* anti-arthritic studies of ellagic acid from *Kirganelia reticulata* baill and its molecular docking. *J Appl Pharm Sci* **2014**, *4*, 24-31.
17. Tanideh, N.; Nazhvani, S.D.; Bahrami, A.; Mehrabani, D.; Akbarzadeh, A.; Azarpira, N.; Lotfi, M.; bagheri, M.H.; Koohi-Hosseinabadi, O., The effect of hydroalcoholic extract of *Mangifera indica* on induced osteoarthritis of knee in male guinea pigs. *Comp Clin Path* **2016**, *25*, 973-979.
18. Akhtar, N.; Khan, N.M.; Ashruf, O.S.; Haqqi, T.M., Inhibition of cartilage degradation and suppression of PGE_2 and MMPs expression by pomegranate fruit extract in a model of posttraumatic osteoarthritis. *Nutrition* **2017**, *33*, 1-13.
19. Wen, L.; Qu, T.B.; Zhai, K.; Ding, J.; Hai, Y.; Zhou, J.L., Gallic acid can play a chondroprotective role against AGE-induced osteoarthritis progression. *J Orthop Sci* **2015**, *20*, 734-741.
20. Ding, S.L.; Pang, Z.Y.; Chen, X.M.; Li, Z.; Liu, X.X.; Zhai, Q.L.; Huang, J.M.; Ruan, Z.Y., Urolithin A attenuates IL-1β-induced inflammatory responses and cartilage degradation via inhibiting the MAPK/NF-κB signaling pathways in rat articular chondrocytes. *J Inflamm (Lond)* **2020**, *17*, 13.

21. Shukla, M.; Gupta, K.; Rasheed, Z.; Khan, K.A.; Haqqi, T.M., Bioavailable constituents/metabolites of pomegranate (*Punica granatum* L) preferentially inhibit COX2 activity *ex vivo* and IL-1β-induced PGE_2 production in human chondrocytes *in vitro*. *J Inflamm (Lond)* **2008**, *5*, 9.
22. Ahmed, S.; Wang, N.; Hafeez, B.B.; Cheruvu, V.K.; Haqqi, T.M., *Punica granatum* L. extracts inhibits IL-1β-induced expression of matrix metalloproteinases by inhibiting the activation of MAP kinases and NF-κB in human chondrocytes *in vitro*. *J Nutr* **2005**, *135*, 2096-2102.
23. Haseeb, A.; Khan, N.M.; Ashruf, O.S.; Haqqi, T.M., A polyphenol-rich pomegranate fruit extract suppresses NF-κB and IL-6 expression by blocking the activation of IKKβ and NIK in primary human chondrocytes. *Phytother Res* **2017**, *31*, 778-782.
24. Rasheed, Z.; Akhtar, N.; Haqqi, T.M., Pomegranate extract inhibits the interleukin-1β-induced activation of MKK-3, p38α-MAPK and transcription factor RUNX-2 in human osteoarthritis chondrocytes. *Arthritis Res Ther* **2010**, *12*, R195.
